# Bioengineering Embryonic Stem Cell Microenvironments for the Study of Breast Cancer

**DOI:** 10.3390/ijms12117662

**Published:** 2011-11-08

**Authors:** Nurazhani Abdul Raof, Bridget M. Mooney, Yubing Xie

**Affiliations:** College of Nanoscale Science & Engineering, University at Albany (SUNY), Albany, NY 12202, USA; E-Mails: nabdulraof@albany.edu (N.A.R.); bm0675@albany.edu (B.M.M.)

**Keywords:** stem cell, microenvironment, hydrogel, three-dimensional culture, breast cancer, metastasis, co-culture

## Abstract

Breast cancer is the most prevalent disease amongst women worldwide and metastasis is the main cause of death due to breast cancer. Metastatic breast cancer cells and embryonic stem (ES) cells display similar characteristics. However, unlike metastatic breast cancer cells, ES cells are nonmalignant. Furthermore, embryonic microenvironments have the potential to convert metastatic breast cancer cells into a less invasive phenotype. The creation of *in vitro* embryonic microenvironments will enable better understanding of ES cell-breast cancer cell interactions, help elucidate tumorigenesis, and lead to the restriction of breast cancer metastasis. In this article, we will present the characteristics of breast cancer cells and ES cells as well as their microenvironments, importance of embryonic microenvironments in inhibiting tumorigenesis, convergence of tumorigenic and embryonic signaling pathways, and state of the art in bioengineering embryonic microenvironments for breast cancer research. Additionally, the potential application of bioengineered embryonic microenvironments for the prevention and treatment of invasive breast cancer will be discussed.

## 1. Introduction

According to the International Agency for Research on Cancer, breast cancer is the most frequently diagnosed malignant disease and leading cause of cancer deaths among women worldwide. In 2011, it is estimated that 230,480 women in the United States will be diagnosed with invasive breast cancer and 57,650 with noninvasive breast cancer, from which 39,520 women will die [1]. Despite the fact that the death rate of breast cancer is expected to decrease due to better awareness, early detection and improved treatment, it is anticipated that breast cancer will remain the second leading cause of death after heart disease in the United States.

Breast cancer occurs when cells in the lining of the milk duct or the lobules that provide milk to the duct undergo a series of mutations. These mutations may take several years to develop. The cause of these cellular mutations is still under investigation but may be attributed to several factors including aging, family history, carcinogens, poor diet, smoking and excessive drinking. Breast cancer cells proliferate uncontrollably and may induce metastasis, which is the main cause of death associated with breast cancer [2,3]. Metastasis is the complex process in which cancer cells spread out to other parts of the body through the bloodstream or the lymphatic system [4]. Tumor microenvironments play an important role in the regulation of metastasis [5–8]. The communication between cancer cells and their microenvironments triggers cancer cells to break away from the original tumor and invade other areas of the body [9–12]. Cell migration and invasion play a pivotal role in the onset of metastasis. Current treatments focused on eradicating metastatic cancer cells include systemic therapy (e.g., chemotherapy, hormonal therapy, biological therapy) and local therapy (e.g., surgery, mastectomy, lumpectomy). While advances in science and technology have aided in providing improved treatments to reduce the number of deaths caused by breast cancer, it remains a huge challenge to fully understand the mechanisms of cell migration and invasion, and to completely eliminate cancer cells to prevent disease recurrence and metastasis. The engineering of unique tumor microenvironments *in vitro* which can manipulate the proliferation and migration of metastatic breast cancer cells may permit enhanced study of cancer metastasis. Consequently, this could provide greater insight into the decision-making processes regarding the growth, migration, and invasion of cancer cells and its subsequent prevention.

With the advancement of embryonic stem (ES) cell technology, the use of bioengineered ES cell microenvironments provides an ideal platform to study and understand the inhibition along with the metastatic potential of invasive breast cancer cells *in vitro*. Firstly, the undifferentiated ES cell microenvironment represents a unique microenvironment to counteract tumorigenesis and metastasis. Prior studies indicate that cancer cells generally interact with a microenvironment that facilitates plasticity, tumorigenicity and metastasis [5,9,13–15], while ES cells sustain a microenvironment regulating self-renewal and differentiation [16,17]. The embryonic microenvironment has the potential to reverse the malignancy state of tumors as it might consist of environmental factors that have the ability to reprogram cancer cells into a less invasive phenotype [18–25]. Furthermore, research has indicated that the undifferentiated ES cell microenvironment reduces cancer cell growth, as opposed to, the differentiated microenvironment which increases growth [26]. Secondly, since breast cancer cells are believed to originate from mutations caused by rapidly dividing stem cells, attempts to restore the mutated tumor microenvironment with a bioengineered embryonic microenvironment may overturn cancer cell progression. Thirdly, the creation of ES cell microenvironments may help identify anti-tumor and/or anti-metastasis factors through the study of interactions of ES cells and metastatic breast cancer cells. In addition to discovering the ability of the ES cell microenvironment to halt cancer cell growth and migration, the co-culture of ES cells and breast cancer cells would also offer the recreation of *in vitro* breast cancer models for mechanism studies and drug screening.

In this review, we will summarize findings regarding the utilization of the embryonic microenvironment *in vivo* and *in vitro* to understand and inhibit cancer metastasis. A brief discussion of breast cancer cell and embryonic stem cell characteristics will be included. Lastly, we will discuss the recent discovery within our own laboratory that bioengineered 3D embryonic microenvironments inhibit the proliferation and migration of metastatic breast cancer cells. Together, the study of ES cell-cancer cell interactions in a bioengineered system will provide valuable insight into the fundamental understanding of tumor progression and therapeutic development for metastatic diseases.

## 2. Characteristics of Breast Cancer Cells and Tumor Microenvironments

### 2.1. Uncontrolled Tumor Growth

Excessive cancer cell proliferation is due to the overexpression of proteins produced by oncogenes, which are created via the mutation of normal proto-oncogenes and tumor suppressor genes. Mutated cells do not respond to typical cell cycle regulation mechanisms such as programmable cell death, known as apoptosis, leading to the overgrowth of damaged cells. For instance, proto-oncogenes as well as cell surface receptors, *erb-B2 and* epidermal growth factor receptor (*EGFR)* are normally activated after the binding of the EGF ligand to induce normal cell proliferation. The binding subsequently induces erb-B2 and EGFR endocytosis and regulates the normal intracellular signaling cascade. In contrast, the *erb-B2 and EGFR* oncogenes, which are categorized under the receptor tyrosine kinases family, send signals to promote cancer cell division without having to bind to any growth factors resulting in dramatic, uncontrolled growth of tumor cells. In addition, the overexpression of *EGFR* and erb-B2 oncogenes stimulates invasiveness of breast cancer cells [27].

Other important mutant proto-oncogenes that are responsible for breast cancer cell proliferation and differentiation include cyclins, cyclin dependent kinases (CDK), the tyrosine kinase family of growth factor receptors, and the c-myc oncogene [28]. The mutated/transformed tumor suppressor genes that accelerate the breast cancer cell growth include p53, retinoblastoma (Rb) gene, BRCA1 and BRCA2, PTEN, ATM, Brush-1, Maspin and nm231 [29]. These previously mentioned oncogenes are just a few examples of impaired genes in breast cancer as there are over thousands of reported deviations within the genome [30–32].

### 2.2. Metastasis

In order for metastasis to occur, breast cancer cells must first undergo several critical cascades influenced by genetic or epigenetic modifications. Initially, breast cancer cells proliferate rapidly enhancing their aggressiveness due to the presence of oncogenes. The extracellular matrix (ECM) surrounding breast cancer cells, is subsequently degraded by matrix metalloproteases (MMPs) allowing cells to migrate and invade the stroma. MMPs are a family of proteinases that regulate cell signaling to promote growth, inflammation, and/or angiogenesis [33]. In addition to MMPs, the delocalization of cancer cells from the primary tumor is also caused by the decrement in the expression of cell adhesion proteins, for example, CD44 [34], E-cadherins [35], integrin [36], and vimentin [35]. During this phase, cancer cells in the primary tumor are transitioning in what is referred to as epithelial-mesenchymal transition (EMT), which is essentially a program that induces cells to be highly mobilized in order to migrate away [37,38]. Breast cancer cell migration is guided by chemokines through the paracrine loop, such as CCL18 [39], CCR4 [40], CCL25 [41], CXCL14 and CXCL15 [42]. Additionally, invasive breast cancer cells, MDA-MB-231, undergo metastasis *in vivo* based on the communication between their secreted factors, colony stimulating factor-1 (CSF-1) and EGF, which are growth factors released by surrounding macrophages [43]. Transcription factors involved during the EMT state of breast cancer include Snail, Slug, Twist, Six1, Lbx1, and ZEB [44]. The known signaling pathways that influence the behavior of these transcription factors during EMT are TGF-β, Wnt/β-catenin, and Msx2/Cripto pathways [45]. Moreover, tumor necrosis factor-alpha (TNF-α) is involved in the promotion of metastasis. TNF-α is a transmembrane protein that stimulates tumor proliferation and survival via NF-κB-, PKCα- and AP-1-dependent signaling pathways [46].

The morphological processes of a cancer cell during the EMT phase are termed lamellipodia, filopodia and invadopodia, and are governed by a very active actin-cytoskeletal component and a high concentration of proteases [47,48]. Briefly, lamellipodia are wide protrusions located at the edge of cells during motility, while filopodia are long and thin actin filaments protruding several micrometers ahead beyond the lamellipodia coverage. Invadopodia are structures possessed by cells that are highly enriched in actin filaments and are responsible for degrading the ECM to drive cancer cell invasion. One critical actin-binding protein that facilitates normal cellular migration is Profilin1. It has been reported that the down-regulation of this protein promotes motility in breast cancer cells (MD-MB-231) through the increased amount of lamellipodia [49,50]. Another well-known actin skeletal protein that drives metastasis is Mena. Mena is upregulated and highly expressed in invasive breast cancer cells [51]. Regarding breast cancer motility during metastasis, two types of cancer cell migration exist and are termed amoeboid migration and collective cell migration [52]. Amoeboid migration is defined as the movement of cells that have reduced focal adhesions and maintained high flexibility. Collective cell migration refers to the cell movement while in contact with neighboring cells. Following the migration, highly motile breast cancer cells will move directionally towards blood vessels and enter the blood or lymphatic vasculature via intravasation [53]. As a result the invasive breast cancer cells migrate out of the primary tumor via blood vessels and reach a secondary organ in the body via extravasation, creating a new metastatic site.

### 2.3. Tumor Microenvironment

Breast cancer cell growth and behavior that promotes metastasis is mainly influenced by the complex and highly structured microenvironment [15,54–59]. The tumor microenvironment consists of soluble factors, ECM and neighboring cells [8,60]. Soluble factors include encircling cytokines and growth factors that have the potential to guide cells toward a malignant state. MMPs exemplify soluble factors that modulate the tumor microenvironment [33]. There are several types of MMPs that work synergistically to induce breast cancer metastasis [61]. MMPs function by cleaving cell surface receptors, which in turn, detaches the bound ECM. Consequently, this process leads to the degradation of the ECM that permits breast cancer cells to migrate and intravasate. Prior studies indicate that MMP-1, -2, -8, -9 -10, -11, -12, -13, -15, -19, -23, -24, -27 and -28 promote breast cancer development and tumor progression [62]. In particular, MMP-1, -9, and -13 are strongly correlated with the incidence of breast cancer metastasis and are potential markers for poor prognosis of invasive breast cancer [63–66]. The activity of MMPs can be inhibited by endogenous tissue inhibitor of metalloproteinases (TIMPs), including TIMP-1, TIMP-2, TIMP-3, and TIMP-4 [67]. Among these inhibitors, TIMP-1 inhibits MMP-1, -3, and -9 more effectively than TIMP-2 [68–70], while all types of TIMPs are able to inhibit active MMP-13 [71,72]. Another critical soluble factor that is highly expressed in the tumor microenvironment is EGF [73]. EGF stimulates the proliferation of breast cancer cells by binding to EGFR which is one of the oncogenes of breast cancer. EGF also displays chemotactic properties in facilitating cell migration towards metastasis [74]. Two additional examples of growth factors associated with the development of metastatic breast cancer include fibroblast growth factors (FGF) [75] and transforming growth factor-β (TGF-β) [76].

Within the tumor microenvironment, the ECM constitutes the basement membrane and the interstitial matrix, providing a cushion for cells to grow. The mechanical properties of cancer cells, dictated by external exertion from the surrounding microenvironment, impact the degree of invasiveness as well [77]. The ECM is defined as connective tissues comprised of fibrous proteins and polysaccharides. The bidirectional communication and interaction between cancer cells and the ECM contributes to the progression of metastasis. Major components of the ECM in the basement membrane include collagen, elastin, laminin and fibronectin. The interstitial matrix consists of polysaccharides such as proteoglycans and hyaluronic acid. Breast cancer cells are attached to the ECM and other cells via transmembrane glycoproteins such as integrin and E-cadherin. The loss of these proteins is one of the hallmarks of cancer metastasis [78]. Reportedly, the reduced expression of these proteins is associated with other contributing factors in the tumor microenvironment such as TGF-β [79] and MMPs [80].

Another critical factor in the tumor microenvironment is neighboring cells. Fibroblasts, myoepithelial cells, adipocytes, endothelial cells, and leukocytes are examples of cells that surround breast cancer cells. In addition to providing scaffolds for cancer cells to grow, these cells secrete signals, cytokines, and growth factors that may increase the malignancy of cancer cells [81]. The term tumor microenvironment of metastasis (TMEM) coined by Robinson *et al*. includes three critical cells in the tumor microenvironment: an invasive carcinoma cell, a macrophage, and an endothelial cell. The researchers discovered that the density of TMEM was higher in metastatic patients as compared to primary breast cancer patients [82]. A study by Yizraeli *et al*. reported that following the direct application of an electric field to induce apoptosis in breast cancer cells, the apoptotic event was delayed when these cells were co-cultured with fibroblasts as opposed to breast cancer cells alone [83]. This result illustrates the importance of neighboring cells, fibroblasts in this case, in governing tumor malignancy. Furthermore, considering the fact that surrounding cells play a significant role in directing metastasis, *in vitro* co-culture of breast cancer cells with other cells may increase understanding of tumorigenesis. For instance, MCF-7 breast cancer cell proliferation was inhibited when co-cultured with preadipocytes [84]. Additionally, breast cancer cells cultured three dimensionally in the presence of normal breast fibroblasts displayed reversion of tumor phenotype [85] and formation of ductal structures [86]. Several studies have also revealed the enhancement of tumorigenicity of breast cancer cells when they were co-cultured with adipocytes [87], benign mammary epithelial cells [88], bone marrow stem cells [89] and endothelial cells [90]. Reconstructing a human breast cancer model is indeed crucial and theoretically more physiologically relevant with respect to attaining proper understanding of cell-cell interaction related to metastasis.

### 2.4. Reprogramming Breast Cancer Cells

It is possible to halt breast cancer cell progression under certain conditions. Firstly, it may be accomplished through the arrest of cell proliferative capacity, termed cellular senescence [91], which involves the shortening of telomeres [92] or p53 activation [93]. Secondly, blocking of oncogenes contained in breast cancer cells and their microenvironment may facilitate the overturn of cell proliferation and migration. One great example is the treatment of Trastuzumab, a humanized anti-HER2/neu antibody, to inhibit erb-B2 tyrosine kinase receptor (TKR) oncogenic activity [94]. Another possible blocking mechanism is the arrest of MMP activity. Since MMPs play a critical role in tumor invasion and metastasis through the degradation of the ECM, impeding the enzyme may reduce tumorigenesis. The major inhibitors of MMPs include TIMP1 and TIMP2 [95]. Furthermore, blocking the growth factor receptors involved in promoting breast cancer aggressiveness may also contribute to the anti-proliferative capability of cells [96]. The obstruction of the receptor involved in invadopodia formation, platelet derived growth factor receptor (PDGFR), through its transcription factor, Twist1, prevents breast cancer metastasis [97]. Additionally, targeting cell adhesion proteins such as integrin by creating its antagonist is a promising anti-cancer therapeutic strategy [98]. The blocking of certain oncogenes and signaling pathways may induce apoptosis in breast cancer cells as well [99], leading to potential alternatives in breast cancer therapy.

Considering the fact that the tumor microenvironment has emerged as a significant and vital component that drives metastasis, targeting the breast cancer cell microenvironment may be one of the potential solutions in reprogramming breast cancer invasiveness [100]. In particular, this review focuses on utilizing the embryonic microenvironment to replace the tumor microenvironment in order to reprogram cancer cells into a less invasive phenotype, thereby, reducing the instances of metastasis.

## 3. Characteristics of Embryonic Stem Cells and Microenvironments

### 3.1. Self-Renewal and Pluripotency of Embryonic Stem Cells

ES cells originate from the inner cell mass of the blastocyst which forms a few days after the fertilization process. ES cells are taken from the blastocyst and cultured in the lab *in vitro*. ES cells have the capability to self-renew indefinitely making them a significant source of cell regeneration. Additionally, these cells are unique in that they are pluripotent meaning they have the potential to differentiate into all cell types found within the three germ layers—the ectoderm, mesoderm and endoderm. These remarkable and unique properties make ES cells valuable in areas such as regenerative medicine, drug discovery, and diagnostics.

The indefinite self-renewal of ES cells is controlled by several proteins and genes. Transcription factors Oct4, Nanog and Sox2 play a major role in guiding ES cells to proliferate [101]. The downregulation of these genes causes ES cells to undergo differentiation [102]. In this article, we will focus on the microenvironment of undifferentiated ES cells as they restrict tumor cell growth and metastasis, as opposed to differentiated ES cells which promote the growth of tumor cells [26]. Interestingly, Oct4, which is a potent canonical marker for ES cell pluripotency, is downregulated during the EMT phase of breast cancer cells [103]. This particular finding may also indicate that the loss of Oct4 expression in differentiated ES cells creates a microenvironment favorable to tumorigenesis. In the presence of leukemia inhibitory factor (LIF), mouse ES cells hypothetically release certain chemokines/cytokines and growth factors, including interleukin (IL)-1α, IL-10, IL-11, macrophage-colony stimulating factor (M-CSF), oncostatin M, stem cell factor (SCF), VEGF, CXCL1, 2, 6, and 10, CCL2, 4, 7, and 22, CD 40, MMP-9, and TIMP-1 (listed in Table 1) [104]. These cytokines and growth factors provide signals for ES cells to remain alive and retain the capacity for self-renewal and pluripotency. Additionally, the effects of these soluble factors on breast cancer cells are listed in table 1. Although comparable factors are present, breast cancer cells contain mutated genes that lead to under- or overexpression of these chemokines/cytokines and growth factors. Therefore, different levels are present in the tumor microenvironment compared to the ES cell microenvironment. As shown in Table 1, breast cancer cells secrete much higher level of M-CSF, OSM, MIP-2/CXCL-2, MMP-9 and TIMP-1 than ES cells, which are factors correlated with tumor metastasis.

### 3.2. Embryonic Microenvironments

The embryonic microenvironment plays a crucial role in determining the cellular fate of embryonic stem cells: whether to direct cells to self-renew, proliferate, differentiate, remain inactive or experience death. The general components of the embryonic microenvironment also include soluble factors, ECM and neighboring cells. The ECM regulates ES cell signaling in a spatially-patterned fashion by providing structural support to cells, integrating complex cellular signals, and controlling the distribution and activation of growth factors. To mimic the ES cell microenvironment, mouse [125] and human embryonic fibroblasts [126] are initially incorporated as a feeder layer to maintain ES cells in an undifferentiated state. Matrigel [127] and ECM proteins [128] are employed to support the maintenance of ES cell pluripotency in feeder layer-free cultures. Soluble factors for self-renewal of ES cells include LIF for mouse ES cells [129] and basic fibroblast growth factor (bFGF) for human ES cells [130]. Additionally, insulin growth factor-receptor (IGF-R), erb-B2 receptor signaling, and activin-A are required to maintain human ES cell pluripotency [131].

### 3.3. Bioengineering Embryonic Stem Cell Microenvironments

The microenvironments of ES cells play a fundamental role in providing cells with appropriate signaling to induce cell proliferation, differentiation or death. The bioengineering of ES cell microenvironments *in vitro* may permit the emulation of the heterogeneous and highly complex nature of ES cells, enabling a clearer understanding of stem cell fate decision. In order to recapitulate the native stem cell niche, researchers have exploited several bioengineering methods. For example, to replicate the ECM, various types of scaffolds composed of naturally and synthetically derived polymers, or both, have been engineered. Naturally derived polymers include alginate, gelatin, Matrigel and chitosan. Poly(L-lactic acid) (PLLA), poly(ethylene glycol) (PEG) and poly(ε-caprolactone) (PCL) are examples of synthetic polymers. Researchers have demonstrated that human ES cell pluripotency is maintained when the ES cells are cultured in a 3D hyaluronic acid [132], alginate [133,134], or chitosan scaffold [135]. Specifically, our lab has reported that mouse ES cells encapsulated inside aqueous alginate hydrogel microstrands prefer differentiation towards either a mesoderm or endoderm lineage [136]. A study by Peng S. *et al*. revealed that mouse ES cells cultured on gelatin induces cell differentiation into trophectoderm [137]. Furthermore, the application of synthetic polymers within the field of nanotechnology for stem cell culture is mainly the fabrication of nanofibers. For instance, human ES cells cultured on nanofibrous scaffolds exhibit a greater affinity for hepatocyte [138], neuronal [139] and osteogenic differentiation [140,141]. Additionally, mouse ES cells seeded on PCL nanofibers undergo directed differentiation into adipocyte [142] and neural lineages [143]. It is also worth noting that the main purpose of these synthetic nanofibrous scaffolds is to guide stem cell differentiation. Altogether, these findings are indicative of the manners in which different types of engineered biomaterial scaffolds can produce varying stem cell self-renewal or differentiation potentials. In addition to scaffolds, self-renewal of ES cells is maintained by incorporation of growth factors such as LIF [129] or bFGF [130], which will inherently induce greater cell expansion.

To closely imitate the specific types of biomolecules surrounding ES cells, the field of nanotechnology offers a potential solution through the creation of nanoparticles capable of delivering the necessary growth factors to stimulate stem cell proliferation or differentiation. For example, Tran and coworkers studied the influence of nanoparticles on mouse ES cell viability and differentiated morphology and discovered that mouse ES cells differentiate into different cell types, for example, fibroblast-like cells or embryoid bodies, depending on the concentration of the polystyrene nanoparticles in which they are exposed [141]. In reference to the neighboring cells of ES cells, the emergence of cellular patterning technology has yielded potential techniques for taking advantage of bioengineered ES cell microenvironments comprised of manufactured cell-cell interactions [144]. Specifically, this technology contributes to co-culture feasibility through the creation of a combinatorial library of cells that ordinarily exist *in vivo*. In addition, the possibility of investigating the influences of ES cell location and spatial proximity in relation to other types of cells has become tangible. Most importantly, mouse ES cells maintain their pluripotency after laser direct-write patterning and this particular finding reveals the mechanisms for exploitation of this technology in order to understand cellular interactions between ES cells and breast cancer cells.

## 4. Embryonic Microenvironment and Cancer

### 4.1. The Importance of Embryonic Microenvironments in Inhibiting Tumorigenesis

The importance of the embryonic microenvironment in cancer progression and metastasis has been demonstrated in zebrafish, chick and mouse embryonic models [26,145–147]. Mintz & Illmensee performed the initial study implying that exposure to the mouse embryonic microenvironment reprograms teratocarcinoma cells into cells capable of differentiating into normal cells [148]. Pierce *et al*. showed that embryonic microenvironments inhibit the tumorigenicity of embryonal carcinoma cancer cells [149]. Hendrix and colleagues performed multiple studies demonstrating that embryonic microenvironments prevent human melanoma cells from tumorigenesis after implantation into the embryo of zebrafish [150] or revert the metastatic melanoma phenotype to its cell type of origin in an embryonic chick model [24]. The capability of the embryonic microenvironment to inhibit tumorigenesis and reprogram metastatic cancer cells to a less aggressive phenotype was confirmed using an *in vitro* mouse embryo model [151] and intrauterine transplantation mouse model [152], respectively. These studies reveal the uniqueness of the embryonic microenvironment in halting tumorigenicity. For example, Patton *et al*. observed enhanced tumor growth when cancer cells were implanted in adult zebrafish as opposed to an embryonic microenvironment [153]. These findings are supported by Kasemeier-Kulesa *et al*., who reprogrammed human multipotent tumor cells into a benign phenotype when transplanted in a neural crest chick microenvironment [147]. All this evidence demonstrates the capacity of the embryonic microenvironment to either delay or reverse tumorigenesis, suggesting that embryonic microenvironments might contain factors that could inhibit cancer growth and metastasis.

### 4.2. Convergence of Tumorigenic and Embryonic Signaling Pathways

Embryonic signaling pathways such as Notch, Hedgehog (Hh), Wnt, and TGF-β, are responsible for initiating and driving EMT during embryogenesis [154,155]. These pathways are imperative in maintaining the highly-regulated ES cell processes of proliferation, differentiation, movement and polarity. Tumorigenic and embryonic signaling pathways cross paths during the course of EMT. Additionally, deregulation of embryonic signaling pathways is found in breast, pancreas, and lung tumors [45].

**Figure 1:**
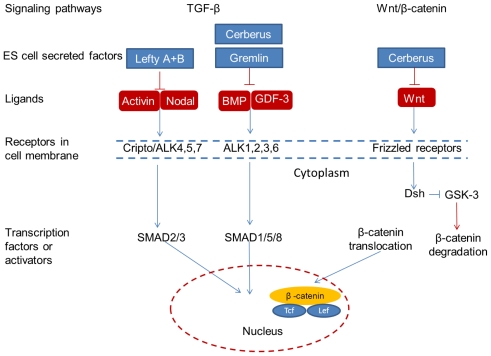
Schematics of TGF-β and Wnt signaling pathways that show the cross-path in tumor and ES cell microenvironments. ES cells can secrete inhibitors of TGF-β and Wnt to regulate the normal embryonic program.

TGF-β and Wnt/β-catenin signaling pathways are misexpressed in breast cancer and associated with poor clinical outcomes [37,38]. The TGF-β pathway activates Notch, Hh and Wnt signaling and plays an important role in the process of EMT, embryogenesis and tumorigenesis. Two main branches of the TGF-β signaling pathway are SMAD1/5/8 and SMAD 2/3. The former branch is mediated by the binding of BMP or GDF ligands to TGF-β receptors ALK1, 2, 3, and 6, whereas, the latter branch is mediated by the binding of Activin and Nodal ligands to TGF-β receptors ALK4, 5, and 7 (Figure 1). The activation of TGF-β signaling triggers the phosphorylation of SMADs which subsequently form complexes with SMAD that translocate to the nucleus and regulate gene expression. In this way, the induction of EMT via cell signaling proteins, SMAD3 and SMAD4, is activated by the TGF-β pathway. Mutations and deregulation of TGF-β receptors leading to inactivation of SMAD4 are observed in various cancers. Examples of TGF-β inhibitors include Lefty A, Lefty B, Gremlin, Cerberus, Follistatin, and Chordin. The mechanism of Wnt signaling involves the binding of a Wnt ligand to the FRIZZLED receptor to activate Dishelved (Dsh) protein (Figure 1). Dsh inhibits GSK-3 which is responsible for β-catenin degradation. Upon Wnt activation, β-catenin accumulates in the cytoplasm and translocates to the nucleus where it interacts with the transcriptional factor T-cell Factor/Lymphocyte Enhancing Binding Factor (Tcf/Lef) [155]. In tumors, Wnt signaling is active and thus stabilizes β-catenin, whose binding to Tcf/Lef activates oncogenes of c-myc and cyclin D1 and stimulates tumor cell proliferation. Documented inhibitors of Wnt signaling include Dkk1, 3, sFRP, WIF-1 and Cerberus.

As previously mentioned, embryonic signaling pathways are tightly controlled in the ES cell microenvironment. Mutations occur within tumor and stromal cells in a tumor microenvironment. Breast tumor cells and ES cells secrete cytokines and chemokines to their microenvironment, however, breast cancer cells secrete a significantly higher level of soluble factors, which is correlated with tumor metastasis (e.g., M-CSF, OSM, MIP-2/CXCL-2, MMP-9, TIMP-1). Undifferentiated ES cells could secrete Lefty A, Lefty B, Gremlin, Cerberus, *etc*., which are inhibitors of TGF-β and Wnt. Tumorigenic and embryonic microenvironments converge via TGF-β and Wnt signaling pathways. However, deregulation of embryonic signaling pathways occurs and critical inhibitors to regulate normal embryonic pathways are missing in a tumor microenvironment. The ES cell microenvironments have great potential to supply critical signaling molecules and reprogram the abnormal embryonic signaling pathway in tumors.

### 4.3. Breast Cancer Stem Cells and Embryonic Stem Cell Microenvironment

The recurrence of breast cancer accompanied with the manifestation of metastasis may be attributed to the presence of breast cancer stem cells (CSCs) within the tumor. Breast CSCs are a small subset of cells coexisting within the breast cancer cell population that have the ability to self-renew and to differentiate into heterogeneous cancer cells. The origin of breast CSCs is not fully understood but is thought to arise from a mutation that occurs during continuous stem cell division and replication. Al Hajj *et al*. are the first researchers to identify a subset of breast CSCs isolated directly from patients [156]. Their study demonstrates that only a small subset of CD44^+^/CD24^−^ breast cancer cells is capable of driving carcinogenesis and possesses the ability to self-renew and to generate multiple cancer cell types. In addition to the CD44^+^/CD24^−^ phenotype, other prominent biomarkers of breast CSCs include aldehyde hydrogenase (ADH)1 [157], CD49f, CD29, and CD133 [158]. Breast CSCs are also highly regulated by their complex and dynamic niche. Bidirectional communication between breast CSCs and their surroundings, such as neighboring cells (fibroblasts, endothelial cells, macrophages, mesenchymal stem cells, *etc*.) and soluble molecules (CXCL12, IL-6, IL-8, *etc*.) greatly influences cellular development. For instance, the cytokines IL-6 and CXCL7 are secreted from nearby mesenchymal stem cells resulting in the self-renewal of breast CSCs via the activation of STAT3/NFκB signaling [159]. Targeting the breast CSC niche may provide an alternative method to eradicate the source of breast cancer in order to prevent disease recurrence or metastasis [160,161]. Several excellent review papers on breast CSCs are referenced for readers interested in obtaining greater detail regarding this subject [45,162,163].

Wong *et al*. elucidated an important aspect in reference to the relationship between breast CSCs and the ES cell microenvironment and reported that the ES cell-like gene module is activated in diverse human epithelial cancers, including liver, breast, prostate, gastric and lung cancer, where identification of an ESC-like signature is a powerful predictor of metastasis and death. In particular, c-Myc, rather than other oncogenes, is sufficient to reactivate the ES cell-like transcriptional program in normal and cancer cells [164]. Alternatively, Somervaille *et al*. implemented a leukemia stem cell model system to study the similarity of CSCs and ES cells. The researchers illustrated that CSCs share transcriptional regulators of self-renewal with ES cells, such as Hmgb3, Cbx5, Mtf2, and Orc21 [165]. However, this ES cell-like state in CSCs is not mediated by the upstream regulators Nanog, Oct4, or Sox2, which are essential transcription factors for maintaining the pluripotency of ES cells. Furthermore, it is predicted that the poor prognosis of human malignancies is caused by an inappropriate expression of upstream regulators linked with a down-stream ES-like program for aberrant self-renewal of ESCs. Therefore, ES cells self-renew in a highly regulated manner, whereas CSCs self-renew in a poorly controlled manner. This study supports the notion that restoring the normal niche of the ES cell microenvironment may cause breast CSCs to convert to a benign phenotype.

Breast CSCs also employ embryonic signaling pathways in order to commence EMT for the occurrence of metastasis. However, unlike embryogenesis, metastasis is driven by transformation of embryonic pathways. Considering the heavy involvement of these altered embryonic pathways in driving metastasis, it is clear that targeting them is a promising approach in treating breast cancer. We hope that replacing the breast CSC microenvironments with a normal ES cell niche may alleviate the deregulation of embryonic pathways, which in turn will restrict metastatic disease.

### 4.4. Reprogramming of Metastatic Cancer Cells Using ES Cell-Conditioned Microenvironments

A state of the art technique for examining the potential ability of ES cell-conditioned microenvironments to cease cancer cell growth involves hES cell-conditioned Matrigel and was performed by Hendrix’s group. This research demonstrated that hES cell-conditioned Matrigel reprograms metastatic melanoma cells to a less aggressive phenotype and significantly inhibits invasiveness and tumorigenesis [166]. Most of these *in vivo* and *in vitro* studies focus on melanoma cells. At this point, only two studies have investigated the effects of human ES cell-conditioned Matrigels on human breast cancer cells. Postovit *et al*. showed that cancer cells overexpress Nodal genes, the embryonic morphogen expressed in hES cells. Basically, these genes are responsible for the maintenance of pluripotency in ES cells and tumorigenesis in cancer cells. The expression of these genes is regulated by Lefty, an inhibitor of the Nodal signaling pathway. However, Lefty is not expressed in breast cancer cells or their microenvironments. As shown in Figure 1, exposure of cancer cells to the ES cell-conditioned Matrigel (containing Lefty) could suppress Nodal gene expression in cancer cells leading to apoptosis, which is significant because recombinant Lefty is unable to downregulate Nodal [23]. This finding is directly attributed to the fact that the ES cell microenvironment-derived Lefty was glycosylated and more physiologically active in comparison to recombinant Lefty. In addition to Nodal knock-down, they also observed the down regulation of VE-Cadherin (a tumor angiogenesis marker) after the exposure of human melanoma cells to hES cell-conditioned Matrigel [167]. Subsequent studies performed by this group have offered additional factors within the human ES cell microenvironment that potentially contribute to epigenetic reprogramming of metastatic tumor cells such as DNA methylation and microRNA regulation [22].

**Figure 2:**
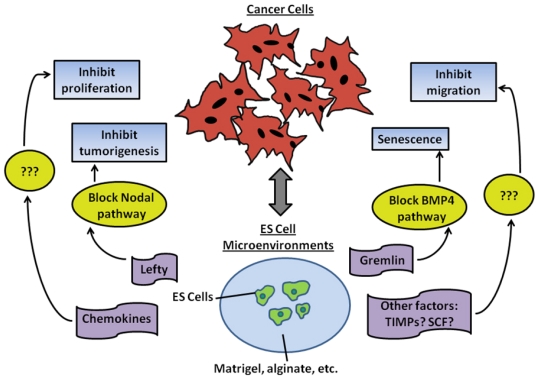
Schematics of the interactions of embryonic stem (ES) cell microenvironments and cancer cells.

Kim *et al*. further clarified the implementation of a mouse ES-conditioned Matrigel to inhibit human melanoma cells. Their findings highlighted the discovery of an important factor in the ES cell microenvironment responsible for halting cancer cell proliferation, Gremlin. Gremlin is an antagonist of bone morphogenetic protein 4 (BMP4) that regulates stem cell expansion and differentiation. As shown in Figure 1, the presence of Gremlin induces cellular senescence in melanoma cancer cells [25]. Another study by Ben-Porath *et al*. revealed that poorly differentiated tumor cells consist of genes that are highly expressed in human ES cells such as Nanog, Oct4, Sox2 and c-myc as compared to well differentiated tumor cells [168]. The relationship between human ES cell conditioned medium and human epithelial, ovarian, prostate and breast cancer cell proliferation was assessed by Giuffrida *et al*. [21]. In their study, cancer cell proliferation decreases after exposure to human ES cell-conditioned media as compared to the controls containing mouse fibroblast conditioned media and normal cancer cell media. Dilution studies demonstrated that depletion of nutrients in human ES cell-conditioned medium does not contribute to the arrest of cancer cell proliferation [21]. This study confirms that hES cells secrete factors smaller than 10 kD which plays a major role in inhibiting cell proliferation and terminating the cell cycle. Referring to Table 1, which contains a list of cytokines released from undifferentiated murine ES cells, it is apparent that chemokines are factors that typically have molecular weights less than 10 kD and play a critical role in inhibiting cancer cell proliferation (Figure 2). These findings implicate ES cell-derived active microenvironments as important sources of tumor suppression factors, which are summarized in Table 2.

### 4.5. Bioengineered Embryonic Microenvironments for Breast Cancer Research

Most of the previously described experiments demonstrated “state of the art” techniques for exploring the embryonic microenvironment and were analyzed to determine if it is possible to arrest cancer cell proliferation and reprogram metastatic cancer cells using *in vivo* microenvironments or ES cell-conditioned Matrigel. In addition, the majority of these studies focused on human metastatic melanoma cells. The limited research related to the exploitation of *in vitro* dynamic embryonic microenvironments in an effort to understand and inhibit breast cancer metastasis prompted further investigation. In our lab, we have explored the possibility of using 3D bioengineered embryonic microenvironments consisting of alginate hydrogel and mouse ES cells to examine the possibility of dynamically reversing malignant growth and migration of metastatic breast cancer cells.

Bioengineered 3D *in vitro* models for studying dynamic ES cell-cancer cell interactions can bridge the gap between 2D cell cultures and whole-animal systems. Cancer cells cultured in 3D alginate hydrogel are capable of forming multicellular tumor spheroids [170–173]. The 3D microenvironment recapitulates the native setting and permits tumorigenesis as compared to 2D [174]. Alginate, obtained from cell walls of brown algae, is an anionic polysaccharide that consists of βD-mannuronate and α-L-guluronate residues [175,176]. In the past, it has been broadly utilized in tissue engineering and regenerative medicine due to its biocompatibility, as well as, gentle gelling behavior. In addition, the ability of alginate to retain a large quantity of water mimics glycosaminoglycans (GAG), which are a component of the ECM. [177,178] Analogous to GAG, alginate is a negatively charged polysaccharide with a high viscosity that could also provide a good platform and structural integrity to allow for smooth cell migration. One method of forming alginate hydrogel is through exposure of the alginate solution to divalent cations such as calcium chloride, barium chloride, and zinc chloride. Cells are encapsulated in alginate hydrogel by combining them with the alginate solution prior to exposure to divalent cations. Alginate hydrogel is further coated with poly-L-lysine to modify its microenvironment from gelled to aqueous via a chelating agent such as sodium citrate. The encapsulation technology employing alginate hydrogel has been successfully performed in studies related to various types of systems such as stem cell differentiation [179–183], pancreatic islets delivery for diabetes treatment [184–187] as well as 3D disease models [188,189].

**Figure 3:**
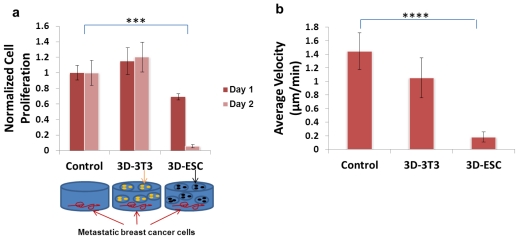
Inhibitory effects of bioengineered ES cell microenvironments on metastatic cancer cells. (**a**) Cell proliferation; (**b**) Cell migration. Mouse ES cells in 3D alginate hydrogel beads (3D-ESC) significantly inhibited the proliferation and migration of metastatic breast cancer cells while NIH 3T3 fibroblasts in 3D alginate hydrogel beads (3D-3T3) did not display the significant inhibitory effect. *** *p* < 0.001. **** *p* < 0.0001.

ES cells were encapsulated in alginate hydrogel microbeads via an electrostatic-driven method. The cells were allowed to grow within the microbeads creating an *in vitro* bioengineered embryonic microenvironment [169]. ES cells in alginate hydrogels remained in an undifferentiated state, which supports their self-renewal and pluripotency. The co-encapsulation of ES cells with metastatic breast cancer cells in aqueous alginate microbeads displayed inhibition of tumor formation as opposed to encapsulation of breast cancer cells alone. Further studies involved the exposure of alginate hydrogel microbeads laden with ES cells to highly invasive breast cancer cells to examine the effects on cancer cell proliferation and migration. ES cells were encapsulated in alginate hydrogel microbeads (about 600 μm in diameter) at initial cell densities of 10^5^, 3 × 10^5^, 5 × 10^5^ and 10^6^ cells/mL alginate. The corresponding cell numbers in each microbead were 11, 34, 56 and 113 cells, respectively. Only one bead containing ES cells was added to metastatic breast cancer cells. Following co-culture for 1–2 days, the proliferation levels of metastatic breast cancer cells was examined. We discovered that microbeads containing 113 ES cells (equivalent to10^6^ cells/mL alginate) displayed the highest level of cancer cell proliferation inhibition on day one, while all types of microbeads inhibited cancer cell proliferation by day two. Therefore, we employed the initial cell density of 10^6^ cells/mL alginate for all future studies. One, two, five or ten microbeads containing ES cells were co-cultured with metastatic breast cancer cells. After two days of cultivation, five microbeads containing the 113 cells/bead exhibited the maximum inhibitory effect on the proliferation of metastatic breast cancer cells. The inhibition rate of cell proliferation was over 90%. In particular, the migration of cancer cells was monitored and analyzed using image J software in the absence or presence of ES cells or NIH 3T3 fibroblasts. To quantify the speed of cell migration, the average velocity was calculated as the mean of all the values of velocity for an individual cell during a 4 hour imaging course. Compared to the control group (metastatic breast cancer cells alone), ES cells in hydrogel significantly restricted the growth and migration of metastatic breast cancer cells while hydrogel microbeads containing NIH 3T3 fibroblasts did not influence these two factors significantly (Figure 3). Based on the results obtained through the measurement of breast cancer cell proliferation and quantification of cell migration after the co-culture, we conclude that mouse ES cells encapsulated in alginate hydrogel microbeads may have secreted soluble factors that inhibit metastatic breast cancer cell growth and migration [169]. This bioengineered embryonic microenvironment provides a new avenue for identifying anti-tumor/anti-metastasis factors to restrict the proliferative capacity and metastatic potential of tumor cells. Elucidation of key components will lead to the creation of bioengineered microenvironments that can restrict metastatic disease progression. Mimicking the embryonic microenvironment to reprogram metastatic cancer cells to less invasive phenotype has great potential to transform the ways in which cancer is treated in the future.

## 5. Outlook

Metastatic cancer cells interact dynamically with a microenvironment that facilitates plasticity, tumorigenicity and metastasis. The unique microenvironment of breast cancer cells plays an important role in the regulation of metastasis. The engineering of cancer cell microenvironments *in vitro* may allow improved study of breast cancer metastasis leading to the discovery of a mechanism that will reverse malignant tumor growth. Metastatic cancer cells and CSCs share aspects of their transcriptional program with ES cells and employ embryonic signaling pathways to drive EMT process. The presence of an ES cell-like gene signature is correlated to poor clinical outcome because the critical molecular messengers are aberrantly expressed in tumor cells and inhibitors are missing in tumor microenvironments. Microenvironments of undifferentiated ES cells have the potential to restrict tumor cell growth and metastasis, as they might supply molecular messengers or inhibitors to regulate or reprogram the abnormal embryonic signaling pathway and restore normalcy. Recent findings from our lab have demonstrated the capacity of a bioengineered embryonic microenvironment to inhibit breast cancer metastasis, proving its potential in identifying anti-tumor/anti-metastasis factors as well as restricting metastatic disease. In the future, human embryonic stem cells and metastatic breast cancer cells or cancer stem cells will be employed in this system as it is more clinically relevant. In addition, it is essential to explore the feasibility of using induced pluripotent stem (iPS) cells to create an *in vitro* embryonic microenvironment. We anticipate that a more in-depth understanding of ES cell-breast cancer cell interactions will lead to the prevention and treatment of breast cancer metastasis.

## Figures and Tables

**Table 1:** Comparable soluble factors secreted by murine embryonic stem (ES) cells and breast cancer cells and their effects on the latter cells.

Name	Molecular Weight (kDa)	C (pg/mL) Secreted by ES Cells [104]	C (pg/mL) Released by Breast Cancer Cells	Effects on Breast Cancer Cells
CYTOKINES	20–45			

IL-10	20	+++	++	Expressed in tumor samples [105] and associated with reduced disease-free survival [106]

IL-11	23	++	+++	Produced by breast cancer cells [107] and linked to poor survival [108]

IL-1α	33	++	+++	Expressed in poorly differentiated, ERα-negative tumors [109]

M-CSF	18.5	+++	++++	CSF-1/CSF-1R autocrine signaling contributed to the invasion phenotype of breast cancer [42]

OSM (Oncostatin M)	28	++	++++++	0.1–100 ng/mL OSM: inhibited proliferation/changed cell morphology [110,111]; 20–50 ng/mL OSM: increased invasive potential [112]

SCF (Stem Cell Factor)	45	++	—	High expression of SCF and SCF-R in normal mammary samples and low in invasive tumors [113]; enhanced activation of the MAPK and PI3K pathways [114]

VEGF	42	+++	+++	Angiogenic effect [115]
CHEMOKINES	<13			
GCP-2/CXCL6	8	++	—	Upregulated in breast cancer cells [116]
IP-10/CXCL10	10	+++	+++	Promote metastasis in a murine model [117]
KC/GROα/CXCL 1	11.3	+++	—	Angiogenic effect [118]
MCP-1/CCL2	11–13	+++	+++	Highly expressed in breast tumor [119]
MCP-3/CCL7	11	++	—	Overexpressed in breast carcinoma patients [120]
MDC/CCL22	8.1	++	—	Involved in breast cancer lung metastasis [121]
MIP-1β/CCL4	7.8	++	+++	Downregulated in breast carcinoma patients [120]
MIP-2/CXCL2	6	+	+++	Highly expressed in bone metastatic breast cancer [122]
OTHERS	>29			
CD 40	43	++	—	Anti-tumor activity in breast cancer cells [123]
MMP-9	90	++++	+++++	Overexpressed in breast cancer cells [64]
TIMP-1	29	+++++	++++++	Inhibits breast cancer cell apoptosis [124]

**Table 2:** State of the art research regarding the utilization of embryonic microenvironments to understand and inhibit cancer metastasis.

Embryonic Microenvironments	Cancer Cells	Effects	References
Zebrafish embryo model	Human metastatic melanoma cells	Support cell survival and division with no tumor formation.	[150]
Embryonic chick model	Human metastatic melanoma cells	Revert the metastatic phenotype to its cell type of origin.	[24]
hESC-conditioned Matrigel	Human metastatic melanoma cells	Induce a melanocyte-like phenotype and significantly inhibit the *in vitro* invasiveness of cancer cells.	[166]
hESC-conditioned Matrigel	Human metastatic melanoma and breast cancer cells	Decrease Nodal expression and inhibit tumorigenesis.	[23]
hESC-conditioned Matrigel	Human metastatic melanoma cells	Decrease VE-Cadherin expression.	[167]
hESC-conditioned Matrigel	Human metastatic melanoma cells	Identify miRNAs up- and down- regulated in reprogramming of melanoma cells.	[22]
mESC-conditioned Matrigel	Human metastatic melanoma and breast cancer cells	Inhibit cell proliferation, decrease anchorage independence and induce senescence.	[25]
hESC-conditioned medium	Human epithelial ovarian, prostate, and breast cancer cells	Inhibit cell proliferation and cell cycle (increased cells in G1 and deceased cells in S and G2/M phase).	[21]
*In vitro* mouse embryo model	Human melanoma cells	Support the melanoma cell migration inside the embryo model in a way reminiscent of neural crest cells with no tumor growth.	[151]
Bioengineered mESC microenvironment	Rat metastatic breast cancer cells	Inhibit the growth and migration of breast cancer cells.	[169]
